# “I definitely cannot afford to be feeling poorly if there’s no need to be”: a qualitative evaluation of antiviral uptake following suspected occupational exposure to avian influenza

**DOI:** 10.1186/s12889-025-21459-3

**Published:** 2025-02-02

**Authors:** Riinu Pae, Lucy Findlater, Richard Amlôt, Fernando Capelastegui, Gavin Dabrera, Clare Humphreys, Jharna Kumbang, Isabel Oliver

**Affiliations:** 1 South West Health Protection Team, UK Health Security Agency, Bristol, UK; 2 Behavioural Science and Insights Unit, UK Health Security Agency, London, UK; 3 Science Group, UK Health Security Agency, London, UK; 4 Health Protection Research Unit in Behavioural Science and Evaluation, University of Bristol, Bristol, UK; 5 TARZET Division, Clinical and Public Health Group, UK Health Security Agency, London, UK; 6 South West Health Protection Team, UK Health Security Agency, Chilton, UK; 7 East Midlands Health Protection Team, UK Health Security Agency, Nottingham, UK

**Keywords:** “Avian influenza”, “Antiviral medication”, “Uptake”, “Adherence”, “Occupational exposure”, “Behaviour”

## Abstract

**Background:**

Growing numbers of people have been potentially exposed to avian influenza (AI), as the United Kingdom has managed the largest and most sustained series outbreaks in recent years. Antiviral medication is recommended for exposed individuals for chemoprophylaxis to reduce the severity of illness and the likelihood of secondary transmission. However, some individuals have been hesitant or declined antivirals. In this study, we aimed to identify the factors affecting the uptake of and adherence to antiviral medication.

**Methods:**

We interviewed 14 individuals occupationally exposed to avian influenza and conducted focus groups with 15 public health professionals involved in advising and arranging antivirals. The data were analysed thematically based on COM-B factors (capability, opportunity, motivation).

**Results:**

Although participants saw avian influenza as a severe disease, most did not consider themselves susceptible to it because they felt safe in personal protective equipment and knew that bird-to-human transmission was rare. The biggest barrier to uptake and adherence was experiencing side effects, especially if these disrupted day-to-day life or work. Participants who took antivirals followed medical advice in a novel situation, had health conditions or vulnerable family members they wanted to protect. As responding to an outbreak was exhausting, easy access to antivirals for those at most risk was considered important for improving uptake.

**Conclusions:**

The factors affecting antiviral uptake were multifaceted. Public health interventions should prioritise those at most risk and address multiple components of behaviour, such as advising how to manage side effects, addressing concerns about long-term usage and providing convenient access to antivirals for those at most risk.

**Supplementary Information:**

The online version contains supplementary material available at 10.1186/s12889-025-21459-3.

## Background

Avian influenza (AI), also known as avian flu or bird flu, is a viral zoonosis that affects wild and domestic birds. There is a risk of infection in humans through direct contact with infected birds or through a contaminated environment [1]. Although human infection is rare, AI can cause severe illness and has the potential to evolve into a new variant that may lead to a pandemic [1]. Since 2021, the UK has experienced the largest recorded outbreak of AI in birds, which has led to millions of captive birds being culled to manage the outbreak [2]. The current risk to human health relates to both the continued high levels of transmission in birds, increasing the opportunity for mammalian and human exposures, and the apparent ability of the successful clade (2.3.4.4b) to cause direct spillover into mammals [3].

The Animal and Plant Health Agency (APHA) manages the animal health response to AI, including testing, depopulation of infected birds and decontamination of premises. Due to the unprecedented scale of the outbreak, growing numbers of people have been exposed to AI and are at increased risk of infection. To reduce the severity of illness, the likelihood of secondary transmission and the risk of human adaptation, the Health Protection Teams (HPTs) at the UK Health Security Agency (UKHSA) recommend antiviral prophylaxis for people exposed to the H5, H7, or H9 subtypes of AI [4]. Until January 2023, antivirals were recommended before and after exposure to contaminated environments or infected birds, irrespective of personal protective equipment (PPE) usage [5]. In addition to offering antiviral prophylaxis, the HPT also contacts exposed individuals daily via phone calls or text messages for ten days from exposure to monitor for symptoms of AI.

As people who encounter infected birds frequently due to their jobs act as an interface between infected birds and communities, compliance with recommended public health measures is important among this group [6]. However, HPTs have encountered hesitancy and refusal when advising antiviral prophylaxis, especially from people with frequent exposure, such as farm workers, APHA staff and contractors. HPTs also have anecdotal evidence of low adherence with antiviral medication among those frequently exposed.

### Antiviral uptake following occupational exposure

The evidence on the reasons for antiviral uptake and adherence in the context of AI is scarce. Only two studies on adherence and no studies on antiviral uptake among people with occupational exposure to AI were found. A Canadian study found that over 70% of exposed people were taking antivirals, but only 44% reported taking them daily [7], highlighting that the factors influencing uptake might be different from factors affecting adherence. Another study reported pre- and post-exposure antiviral prophylaxis adherence levels of approximately 85% among poultry workers in Israel [8]. Both studies included limited information on the reasons for not completing the entire course, so it remains unclear what factors affect uptake and adherence in occupational contexts.

### Antiviral uptake and adherence in seasonal influenza contexts

The antiviral medication prescribed for AI, Oseltamivir phosphate (Tamiflu), is also commonly used for the treatment and prophylaxis of seasonal influenza. Previous studies on seasonal influenza have linked antiviral uptake and adherence with favourable attitudes to influenza prevention and knowledge about influenza and antivirals, as well as perceiving the disease to be severe and antivirals effective [9–11]. The most common reasons for non-adherence have been side effects and the fear of developing these [12]. Whilst informative, these studies involved the general public who are less likely to be offered antivirals, and looked at seasonal flu, which is more transmittable and generally less severe in humans than AI.

### Behaviour change

The COM-B model of behaviour change has been widely used to understand barriers and facilitators associated with health protective behaviours, including vaccination and medication uptake and adherence [13]. According to the COM-B model, one needs to have the capability, opportunity and motivation to perform a behaviour [14]. COM-B is also part of the Behaviour Change Wheel intervention development framework, which links barriers to intervention options in an evidence-based and systematic way. However, as COM-B is a general model applicable to many behaviours, it benefits from being supplemented with a more context-specific theory. Protection Motivation Theory is commonly used in infectious disease outbreak and emergency response contexts because it explains what motivates people to act in response to a threat [15]. According to this theory, individuals are likely to perform a protective behaviour if they assess themselves to be vulnerable to a hazard that is severe (threat appraisal), consider themselves able to perform the protective behaviour, and believe that the protective behaviour will mitigate the risk effectively with a low response cost (coping appraisal) [16]. Applying COM-B and Protection Motivation Theory together in the current context enables us to explore risk perception-related and broad-ranging factors affecting uptake and adherence to antivirals in detail.

Taken together, as the factors affecting uptake of and adherence to AI-related antivirals in occupational contexts are not well understood, this research aimed to address the gap in the literature and support the public health response. We applied two behaviour change theories to understand the factors associated with medication uptake and adherence to antiviral medication among people potentially exposed to AI as part of their job.

## Methods

### Design

This qualitative evaluation involved semi-structured interviews with people exposed to AI at work. We also conducted focus groups with UKHSA public health professionals who advised and arranged antivirals. The study design and analyses are reported according to the consolidated criteria for reporting qualitative research guidance (COREQ) [17].

### Participants

#### Exposed individuals

The interview participants were identified from the UKHSA case management system (HP Zone). Sampling was purposeful to include participants with various roles, organisations, regions, exposure situations, and people who accepted and declined antivirals.

Fourteen people who were advised to take antivirals following AI exposure at work were interviewed. Of the fourteen participants, eight were female and six were male (Table 1). Four took antivirals during their most recent exposure, five had taken antivirals previously but not during their most recent exposure, and five had never taken antivirals following their AI exposure. Participants lived in different regions of England and undertook various roles. Despite considerable efforts to do so, we could not interview farm workers, people who had consented via intermediaries or APHA contractors who catch and cull infected birds.

**Table 1:** Characteristics of interviewed exposed individuals

Participant characteristics		Number of participants
**Gender**	Female	8
	Male	6
**Age**	18–34	3
	35–64	10
	65 years and over	1
**Antiviral use history**	Took antivirals during their last exposure	4
	Previously took antivirals but not during their last exposure	5
	Never taken antivirals	5
**Region of residence**	East Midlands	5
	South West	3
	East of England	2
	South East	2
	North West	1
	Yorkshire & Humber	1
**Job role**	Animal Health Officer, APHA	7
	Staff, animal welfare charity	2
	Vet, APHA	1
	Participant, Livetec (APHA contractor)	1
	Ranger, Nature Reserve	1
	Sergeant, Police	1
	Landowner-Farmer	1
**Incident type**	Poultry	9
	Wild bird	5

#### Public health professionals

Focus group participants were identified through UKHSA Consultants leading the AI response in regional HPTs. Using convenience sampling, public health professionals who worked on AI incidents and expressed interest were invited to participate.

Fifteen UKHSA public health professionals participated in four focus groups. Two focus groups were conducted with Consultants (five in one focus group and four in another) and two with Health Protection Practitioners (HPP) (three in each focus group). Participants worked in seven regions of England, although public health professionals from all nine regions were invited to participate.

### Materials

The interview guide was based on the COM-B model [14] and Protection Motivation Theory [18]. These theories enable systematic identification of barriers and facilitators associated with protective behaviours, including motivation, capability, opportunity, and risk perception. The interview guide also included questions on how the participants perceived their interactions with HPPs (Supplementary Materials File 1).

The focus group topic guide included questions on public health practitioners’ experiences advising antivirals to exposed people, perceived barriers to arranging antivirals, and how to improve UKHSA response related to antivirals (Supplementary Materials File 2).

### Data collection

UKHSA focus group participants, APHA staff, and their contractor were recruited through their respective organisations via email. The remaining participants were contacted by their local HPT by a telephone call, invited to participate and provided with an information sheet by email. All participants provided informed consent via an online form before participation. The interview participants were informed of their right to withdraw their data up to 1 week after the interview.

Interviews and focus groups were held remotely via Microsoft Teams; to the interviewer’s knowledge, no one else was present during the discussions. Data was collected between September and December 2022. The interviews lasted between 16 and 50 min and the focus groups lasted between 51 and 57 min. The interview participants were compensated with a £20 voucher in line with the National Institute for Health Research guidance [19]. The focus group participants did not receive compensation.

Given the focused aims of this study, its exploratory nature, the depth and relevance of the data collected, and the application of established theories, we stopped recruitment when the data was deemed to have sufficient “information power” (i.e., when we were satisfied that the data provided a comprehensive and new understanding of the topic). This approach allows prioritising the richness and applicability of insights over the mere emergence of new themes (i.e., “data saturation”) [20].

Discussions were facilitated by the first author (RP), a Behavioural Scientist, who at the time of data collection was based in an HPT at UKHSA. She holds a BSc in Psychology, an MSc in Behaviour Change and had some experience in qualitative data collection. She had not met the interview participants before the interviews, but had had limited interactions with four public health professionals prior to the focus group discussions. It was emphasised at the start of the focus group discussions that the facilitator had no particular views on antivirals and that the views expressed will be kept confidential.

### Ethics

The UKHSA Research Ethics Governance Group (REGG) was approached prior to data collection. The REGG confirmed that a review was not required because the evaluation involved participants by virtue of their professional roles and did not include sensitive or upsetting content.

### Analysis

Interviews and focus group discussions were audio-recorded and transcribed verbatim; no field notes were made. The interviewer (RP) conducted a qualitative analysis of the data following a six-step thematic analysis process [21]. NVivo 12 was used to code and develop the themes. Barriers to and facilitators of antiviral uptake and adherence were coded deductively and assigned to a COM-B component. Data on interactions between exposed people and public health professionals were analysed inductively. Analyses were not shared with the participants. Recommendations for practice were developed using the Behaviour Change Wheel framework [14], whereby COM-B components were linked to intervention functions (the broad categories of behaviour change interventions).

## Results

The findings are divided into three sections (see Table 2 for a thematic map). The first section explores the influence of interactions between public health professionals and exposed individuals on antiviral uptake. The second section categorises factors affecting antiviral uptake using the COM-B framework. These factors include capability (knowledge of AI, antivirals, and risk mitigation), motivation (following advice in a novel situation, conducting own risk assessments, feeling safe in PPE, impact of side effects, personal choice depending on individual circumstances, concerns about long-term use, exhausting outbreak management, and potential future uptake), and opportunity (social influences, access to antivirals, and contraindications). As side effects were the only factor affecting adherence and were interlinked with uptake, these were included in the theme on the impact of side effects. The final section includes qualitative evidence of non-adherence with antivirals.

**Table 2:** Main themes of the findings

Research question	COM-B component		Findings
**1. Interactions between public health professionals and exposed persons**	Not linked to COM-B		• HPT contact was reassuring for those with infrequent exposure • HPT contact was seen to be repetitive for those with frequent exposure • HPTs preferred on-site contact due to difficulties contacting exposed individuals • Public health professionals preferred contact through an intermediary over no contact • Need for accessible and engaging materials
**2. Factors affecting uptake of antivirals**	**Capability** The capacity to engage in the behaviour	**Psychological** Capacity to engage in necessary thought processes	• Exposed individuals had varied knowledge of avian influenza, limited knowledge of antivirals, and in-depth knowledge about risk mitigation
	**Motivation** Brain processes that energise and direct behaviour	**Reflective** Evaluation and plans	• Exposed individuals reported following public health advice during their first exposures • Exposed individuals conducted own risk assessments • Feeling safe in PPE influenced risk perceptions • Taking antivirals was seen to be a personal choice • Widespread concerns about long-term use among exposed individuals and public health professionals • Exposed individuals were open to using antivirals in the future if they become more vulnerable, the virus changed or when strongly advised by a health professional • Side effects were the biggest barrier to uptake
		**Automatic** Emotions and impulses	• Exhausting outbreak management necessitates easy access to antivirals
	**Opportunity** Factors lying outside the individual that act as barriers or promoters of behaviour	**Social** Cultural milieu that affects what we think about things	• The role of social influences on antiviral uptake was not clear
		**Physical** Physical opportunity in the environment	• Easy access to antivirals was an important facilitator (e.g. close to infected premises, at a community pharmacy)
			• Contraindications can be a barrier
**3. Non-adherence with antivirals**	**Motivation** Brain processes that energise and direct behaviour	**Automatic** Emotions and impulses	• Side effects were the biggest barrier to adherence
	Not linked to COM-B		• Evidence of frequent non-adherence with antivirals among exposed individuals

## Interactions between public health professionals and exposed persons

People exposed to AI were contacted by a Health Protection Practitioner (HPP) who informed them about their exposure, gathered information for risk assessment (e.g., use of PPE), and provided information and advice, including antiviral prophylaxis and monitoring, when indicated.

Those exposed to AI infrequently or for the first time appreciated the sense of security offered by having contact with an HPP. On the other hand, many of those exposed frequently found the contact to be repetitive and occasionally bothersome. A few of them sought respectful dialogue where they were encouraged to ask questions and get answers about benefits and disadvantages of antivirals. Likewise, public health professionals were concerned that excessive contact might harm trust in UKHSA and make it more difficult to elicit protective behaviours in the future.

HPPs often found it challenging to get hold of exposed individuals via phone, acknowledging that people responding to an outbreak could not answer phone calls in full PPE. Similarly, most of the APHA staff felt that they were contacted at an unsuitable time, with suitable times differing for everyone. HPPs found it more effective to provide antivirals on site in person because doing so enabled them to get hold of exposed individuals quickly and facilitated relationship building:

*“It makes it more personal*,* and that was something I found particularly with the farmers. This is their livelihood*,* isn’t it*,* and they’ve just lost everything. They’re getting 100 phone calls a day from the accountant*,* from Defra*,* from lots of different people. We’re just another faceless people that are bothering them trying to get them to take medication and they don’t have time to speak with us. So that is quite a good way to develop a relationship better*.” – Senior HPP 1, HPP focus group 1.

Some exposed people had contact with public health professionals through an intermediary. Often, these individuals were APHA subcontractors with non-English language preferences who, in some cases, did not have a UK phone number. HPPs expressed concern but reflected that communication and consent via intermediaries was better than no contact. An APHA contractor who worked with people with non-English language preferences highlighted the importance of having information sheets in relevant languages, such as Romanian, Bulgarian, Polish and Russian, and in engaging video and easy-read formats.

## Factors affecting the uptake of antivirals

### Capability

#### Knowledge of AI

Knowledge of AI in humans varied; however, all interviewees knew that whilst AI is very rare in humans, it can cause serious illness and death. Most knew the main symptoms; the few who could not tell specific symptoms knew that AI causes flu-like respiratory symptoms. Most knew that AI is transmitted through close contact with infected birds or when the birds are in contaminated environments. Multiple interviewees mentioned that extremely close contact in a confined environment without wearing PPE was needed for AI to be transmitted to humans. A few of those who did not take antivirals contrasted their exposure to the exposure circumstances of previous AI outbreaks or the 2021 human case in England.

A few were aware that AI could have the potential to cause a pandemic if it changes, resulting in a new strain that is transmissible among humans. Interestingly, interviewees with nuanced knowledge about bird-to-human and human-to-human transmission tended to decline antivirals when offered. One interviewee explained their reasoning:

*“Take these antivirals*,* it will keep you safe. Well*,* they’ll keep you safe from what? From a disease that hasn’t yet consistently learned to jump from human to human or even from bird to human.”* – Animal Health Officer 4, APHA, not taken antivirals.

#### Knowledge of antivirals

Knowledge of antivirals was generally limited, although most interviewees knew that antivirals were recommended to reduce the severity of illness. Some knew that antivirals reduce the chances of transmission. Only a few interviewees had been advised how to minimise side effects. A few interviewees held misconceptions that antivirals offer long-lasting protection or cannot be used for treatment. Interviewees also acknowledged that whilst they would receive more information about antivirals if they asked, they had received no or limited information about antivirals from their employer.

Multiple interviewees believed that taking medicine was unnecessary if they were feeling well, supported by discussions in HPP focus groups. One participant explained, “*I was perfectly well during that period. I didn’t have COVID and I didn’t have H5N1*,* and therefore I didn’t take any antivirals*.” (Landowner-farmer, not taken antivirals). This was evidenced by a few who had taken a prophylactic dose of antivirals as a precaution when they had symptoms of respiratory illness or were “not feeling 100%”, suggesting a limited understanding of chemoprophylaxis.

#### Knowledge about risk mitigation

All the interviewees described in detail how they minimised the risk of transmission without the interviewer’s prompts. APHA staff and contractors working with captive bird outbreaks explained that they followed strict risk mitigation procedures and wore full PPE, including a power hood. Interviewees involved in wild bird incidents described minimising the number of people exposed to AI, wearing available PPE, standing downwind and double bagging the carcasses, among other risk mitigation measures. Most of the participants stated they would contact their GP, UKHSA HPT and/or their occupational health team if they developed symptoms.

### Motivation

#### Following advice in a novel situation

The novelty of AI was a frequent reason for taking antivirals. During their first exposure(s), most interviewees followed public health advice that recommended antivirals: “*If that’s the advice*,* it must be for a reason. So*,* I thought yes I think I should have it.*” (Vet, APHA, took antivirals). Those with continuous long-term exposure often stopped taking antivirals because they gained more knowledge about AI and became more confident that biosecurity measures kept them safe. An interviewee explained why the “sense of the unknown” dissipated over time:

*“At the start of it you’re really concerned about bird flu. Oh my God*,* bird flu*,* really bad. And then as you gain more experience around it*,* you gain more understanding it’s not necessarily the disease in humans*,* but the disease in birds and how it spreads. […] You get more confidence in your kit*,* your PPE. You get more confidence in your colleagues telling you if there’s anything wrong with your PPE*,* or telling you*,* okay*,* you’ve done this*,* phone UKHSA.”* – Animal Health Officer 7, APHA, took antivirals previously.

#### Conducting own risk assessments

There was strong evidence that interviewees undertook their own risk assessments by considering their exposure situation, protective measures, and the benefits and disadvantages of antiviral medication. Most interviewees risk assessed each exposure situation separately, whereas a few had developed fixed views. This was particularly evident amongst those frequently exposed to AI:

*“There is a risk with contracting any virus. It’s whether the risk outweighs everything else. And for me*,* the risk of potentially contacting bird flu is lower than experiencing the side effects of Tamiflu.” –* Animal Health Officer 6, APHA, took antivirals previously.

*“I think the risk is pretty low. I think there’s only been one case in this country of somebody contracting avian influenza and that I believe the chap was sleeping with his ducks*,* which if you sleep with the ducks*,* you’re going to potentially contract things other than that*,* E. coli*,* salmonella etcetera*,* never mind bird flu. And we are pretty well protected*,* we’ve got the equipment. You’ve got to use your initiative and use it properly*,* but it’s all there provided*,* so I think the risk is low and that’s my take on it.”* – Animal Health Officer 3, APHA, took antivirals previously.

#### Feeling safe in PPE

Whilst all the interviewees had had close contact with infected birds, most felt protected by the PPE and the biosecurity procedures they had followed during their exposure, which was strongly echoed in the public health professional focus groups. Notably, for those who felt their organisation invested significantly in PPE and safe disposal of carcasses, the cost of risk mitigation measures was seen as evidence that they already did enough to keep them safe. APHA staff and contractors frequently compared their strict PPE to farm workers who did not develop symptoms despite having had significant unprotected exposure. An APHA interviewee described why they felt “overly safe”:

*“We’re wearing two paper suits*,* two pairs of gloves*,* a hood with filtered airflow. When you’re comparing it to the owners that have been in a day prior to*,* when they’ve been wearing absolutely nothing*,* then it’s just a bit like*,* okay*,* well*,* they’re fine.”* – Animal Health Officer 7, APHA, took antivirals previously.

However, not everyone thought that PPE alone was sufficient to keep them safe:

*“You can’t always guarantee that your face mask has the correct seal*,* or you might rip your glove or something. Or you’ve been handling the bird in an area*,* and you might return to that area*,* perhaps without your full PPE on because there’s no longer a bird in the area*,* but there’s still possibly dander or faeces or something like that. So*,* yes*,* PPE does protect you*,* but it’s not 100% protection.” –* Participant 1, animal welfare charity, took antivirals.

#### Impact of side effects on uptake and adherence

Prior experience with side effects was the most significant barrier to uptake, whereas previous positive experiences with other therapeutic antivirals encouraged uptake. Approximately half of the interviewees who took antivirals had developed side effects, which they attributed to antivirals. As a result, most of them stopped the course prematurely, especially if their daily life or work was disrupted, and declined antivirals during their subsequent exposures.

Interviewees described experiencing nausea, vomiting, headaches, dizziness and flu-like symptoms, making some of them very unwell:

*“I’ve farmed all my life*,* I’ve had two children*,* nothing has ever made me that ill. I had to sleep on the bathroom floor that night with my head over the top of the toilet.”* – Animal Health Officer 1, took antivirals previously.

*“I’ve never felt so poorly in my life*,* and I stopped taking them and I felt a lot better. I’ll be totally honest*,* I didn’t finish the course*,* I felt absolutely rubbish. And I’d got work to do*,* we’re really busy at the moment and I’ve got a child*,* I’ve got horses*,* I’ve got responsibilities*,* and I definitely cannot afford to be feeling poorly if there’s no need to be.”* – Animal Health Officer 6, APHA, took antivirals previously.

Some who experienced side effects nevertheless finished the course. For one interviewee, the severity of AI outweighed mild side effects. The others thought that medication should be taken as prescribed and likened antivirals to antibiotics. An interviewee who shared this opinion but did not finish the course reported feeling guilty:

*“If a medical professional’s telling you*,* you should take them*,* you should take them. But if they’ve made you incredibly ill and you don’t want to be ill*,* and you don’t take them… I felt guilty for not finishing them*,* if I’m honest.”* – Animal Health Officer 1, APHA, took antivirals previously.

Furthermore, APHA staff and their contractor also expressed a sense of professional duty to perform their service for the benefit of the farming community and infected birds. In their view, taking antivirals hindered outbreak management because side effects stopped them from working or made it unsafe:

*“The sooner we get on site and start culling animals it’s better for the animals and the farmers. They only get paid for stock that we cull. So if we haven’t got enough staff to cull this stock then people are losing money.”* – Animal Health Officer 2, APHA, took antivirals previously.

*“… working around gas and machinery and lots of dangerous things*,* anything that makes you not think as clearly or as straight… You’re very reluctant to take it.” – Participant*,* Livetec*,* took antivirals previously*.

#### Personal choice depending on individual circumstances

With a few exceptions, interviewees felt they had the necessary information and were supported in making an informed choice based on their specific exposure circumstances. In general, taking antivirals was considered a personal choice – an optional, not a “must”. A few interviewees took antivirals because they either had a health condition that made them more vulnerable or wanted to protect vulnerable family members: *“I was visiting my family*,* so I wanted to make sure I was protected and not taking anything to them.”* (Veterinary Nurse, animal welfare charity, took antivirals).

#### Concerns about long-term use

There was widespread concern about the long-term use of antivirals. Both focus groups with public health professionals and interviewees with regular exposure to AI were particularly concerned about the continued efficacy of antivirals if they were not used prudently:

*“Say I’d done 25 (infected premises) and taken 25 lots of antivirals*,* is it still going to be as effective on the 26th one if I was to contract something?”* – Animal Health Officer 2, APHA, took antivirals previously.

Many were sceptical about the necessity of taking antivirals continuously for long periods. This apprehension was shared by public health professionals, those who took and those who did not take antivirals:

*“If I’m supposed to take the antivirals for ten days and I get a bird dying every ten days*,* do I then take a course of antivirals? And then do I take another course of antivirals? And then do I take another course of antivirals?”* – Landowner-farmer, not taken antivirals.

*“When they called me for the second time I was surprised. I said*,* well look*,* I just finished very recently one box. Do I really need to carry on? And I believe they actually said*,* oh*,* I will let you know. They called me back*,* so probably they asked someone else*,* and then they told me*,* yes*,* you should have another. It’s the safest thing to do or whatever. Fine*,* I will take it*.” – Vet, APHA, took antivirals.

Similarly, a few interviewees and public health professionals were concerned about the safety of extended use. One interviewee also questioned whether antivirals inadvertently weakened the immune system:

*“I think about future resistance*,* that like with antibiotics being overprescribed*,* it then affects our gut and then we don’t have good healthy gut bacteria*,* so that makes you more vulnerable because your immune system is then compromised.”* – Ranger, Wildlife Trust, not taken antivirals.

#### Exhausting outbreak management

The response to AI outbreaks involved multiple agencies working at pace. The interviewees often described the outbreak management environment as highly stressful and overwhelming, which the public health professionals empathised with. In particular, APHA staff described working long hours, frequent travel and being away from home for several days to weeks. Multiple APHA staff said that arranging, picking up and claiming back the prescription charge for antivirals was “the last thing they wanted to do” on their time off, stressing the importance of making antivirals easy to access.

Interviewees from animal welfare charities also highlighted the emotional distress caused by being requested to cull birds they had been previously cared for. One described the whole site cull as the worst thing that can happen in their industry and contemplated whether their colleagues linking this traumatic experience with the public health calls led to some of them not taking antivirals and eventually blocking the phone number. A few public health professionals noted similar unintended consequences among people who had to cull their non-commercial backyard flocks due to an outbreak.

#### Potential future uptake

Most of the interviewees were receptive to taking antivirals in the future. Notably, many intended to take antivirals therapeutically if they developed symptoms of AI or if the strain became more transmittable to humans. Some interviewees thought they would take antivirals if they were strongly advised by medical or public health professionals, especially if they already had or were to develop health conditions that would make them more vulnerable to serious illness. A few interviewees were also receptive to starting antivirals if they had significant exposure without PPE or had a PPE breach, if their close contacts had symptoms, or if the strain caused more severe illness in humans. However, a few interviewees with strong negative opinions on medical interventions were sceptical about taking antivirals in the future, irrespective of the exposure circumstances.

A participant who had previously declined antivirals summed up when they would consider taking antivirals in the future:

*“If there was some reason that something changed*,* and it was particularly virulent. If I was advised to*,* and people were becoming very ill*,* or somebody said somebody on your site has contracted bird flu*,* we strongly advise that you take antivirals*,* then I would.”* – Animal Health Officer 5, APHA, not taken antivirals.

### Opportunity

#### Social influence

Many interviewees described a “rumour mill” involving stories about negative experiences with antivirals: *“You hear stories where people have not been able to control their bowels and all sorts of things. So that can put people off. […] I felt lucky I was only sick. Could have been worse.” (*Animal Health Officer 1, APHA, took antivirals previously). For some, these stories confirmed their own decision not to take antivirals, although most reported not feeling affected because they considered taking antivirals their decision personal. Nevertheless, public health professionals shared anecdotal examples where hearing stories about others’ experiences with Tamiflu impacted antiviral uptake:

*“It seemed like everyone on that particular site didn’t seem to want the antivirals*,* whereas that wasn’t the pattern that we’d seen in other places. I think sometimes*,* even if they haven’t taken them themselves and got antivirals*,* if someone else has said to them*,* I took some and got terrible side effects*,* then it puts them off even trying.”* – Advanced Health Protection Practitioner, HPP focus group 2.

Although the interviewees did not believe themselves to be influenced by others’ behaviour, there appeared to be a link: those who did not take antivirals had colleagues who did not take antivirals, whereas those who took antivirals also thought that most of their colleagues took antivirals.

#### Access to antivirals

Antivirals were generally received within the day of prescription, which interviewees were pleased about. Interviewees and focus group participants described different ways they had accessed or arranged antivirals: provision close to infected premises or staff bases, home delivery, and collection from a community or hospital pharmacy.

In line with the exhausting outbreak management environment, most interviewees considered on-site provision or home delivery to be the most convenient, followed by self-collection from a community pharmacy. The importance of easy access for improved uptake was also strongly supported by focus group discussions with public health professionals. An interviewee explained their preference:

*“As easy as possible. I’d rather a doctor come to site and give me them. Rather than having to spend my own time going to a chemist when we might be away for two or three weeks*,* you know what I mean? It’s a long old haul*,* and the winter waiting in a chemist you’re almost putting yourself at more risk.” –* Participant, Livetec, took antivirals previously.

Public health professionals also preferred on-site provision, especially for large outbreaks. They often struggled to contact exposed individuals, so travelling to infected premises enabled them to get hold of exposed individuals and build relationships. This anecdotally led to higher antiviral uptake:

*“We had 50-odd people*,* and they’re in PPE 7 am until 7 pm*,* they’re not answering their phones. So we went on to the site and stayed in the clean area*,* and we had the GP on site with us to do antiviral prescriptions. […] In person I think you can really get a grasp of how well they understand it. They can ask you questions over the phone*,* but you can just read their body language*,* can’t you*,* and be like*,* right you’ve understood this or you’re not interested in this. […] It was really*,* really effective. The uptake was much*,* much better*.” – Senior HPP, HPP focus group 1.

Outside the influenza season, the stock of antivirals was limited mostly to hospital pharmacies. Collection from a hospital pharmacy was the least preferred option by both interviewees and public health professionals because it often required considerable travel. Long travel times (40–90 min one way) were a barrier, particularly in rural areas. HPPs also noted that some people, especially temporary migrant workers, did not have access to a personal car and were therefore not able to collect antivirals unless provided on site.

Some interviewees reported that they had to pay for their prescription, which they could claim back from their employer. Although those with favourable opinions on antivirals did not consider this a barrier, a few interviewees and public health professionals found it disappointing.

#### Contraindications

None of the interviewees mentioned having contraindications to antivirals. One interviewee was asked to take a pregnancy test but did not consider this a barrier as she had favourable opinions about antivirals.

### Non-adherence to antivirals

Interviews with exposed individuals and discussions with HPPs evidenced frequent non-adherence: those exposed frequently often had leftover antivirals at home, indicating they had not finished the course as prescribed. A few interviewees mentioned they had kept unused antivirals just in case they developed symptoms of AI, and only one interviewee reported returning an unfinished course to the pharmacy. As side effects were reported to be the only factor affecting adherence, this data was discussed in the corresponding section on factors affecting uptake.

## Discussion

To our knowledge, this is the first study to explore the factors affecting the uptake of and adherence to antiviral medication among individuals exposed to Avian Influenza (AI) at work. Our qualitative analysis identified wide-ranging factors contributing to antiviral uptake, such as knowledge about AI and antivirals, feeling safe in PPE, experiencing side effects and access barriers. In this section, we will consider the implications of these findings and develop recommendations for improving antiviral uptake and adherence among those currently at most risk and to inform the public health management of AI.

Participants who had been exposed to AI saw it as a severe infection, knew its symptoms in humans and whom to contact if symptomatic. They had strong knowledge of transmission risk mitigation and felt well-protected by strict PPE and biosecurity measures during exposure. This is not surprising, considering that the participants were professionals who routinely risk assessed AI situations, contrasting low levels of general knowledge of AI among ‘backyard’ poultry keepers [22]. These findings indicate that individuals exposed to AI frequently were competent in managing their risk with minimal support from the HPT. However, there was a discrepancy in the level of support they felt they needed and received, with some finding the public health response to be out of proportion to their perceived susceptibility to AI. Notably, the interim UKHSA guidance introduced shortly after the data collection for this study ended no longer recommended prophylactic antivirals to individuals who wore PPE without breaches [23], aligning with guidance issued by the Centres for Disease Control and Prevention in the United States [24] and retained as an interim guidance for the 2023/24 season [25]. Nonetheless, the perception among our participants that the public health response was disproportionate to their risk exposure might have already affected trust in UKHSA.

Another possible explanation for our participants not feeling susceptible to AI despite their direct exposure could be a reduction in the perceived likelihood of contracting AI following exposures without negative consequences. This perspective is supported by the finding that some participants followed public health advice during their first exposure(s) but declined antivirals after subsequent exposures. Our findings are consistent with the risk perception literature suggesting that unfamiliar risks are perceived to be more severe and that risks that people believe can be personally controlled – such as by wearing PPE – are considered to be more acceptable (e.g [26]). Lower perceived risk leading to reduced protective behaviours has been observed in other occupational contexts where a risk changed or was perceived to have changed as circumstances changed [27–29]. If our participants stopped taking antivirals after repeated exposures without negative consequences, we might also expect a gradual reduction in other protective behaviours, such as suboptimal PPE usage or adherence to biosecurity measures. However, we did not explore other behaviours in our study, so further research is needed to understand to what extent risk perceptions change and affect protective behaviours such as antiviral uptake and PPE usage over time.

Whilst lower perceived risk could have influenced the decision not to take antivirals, we found side effects to be the key barrier to uptake and adherence, which is consistent with previous studies [9, 10, 12, 30]. About half of our participants who took antivirals experienced side effects, which is in line with a study with Australian healthcare workers [30], but considerably higher than the rates reported in other studies where 1.5–13% reported gastrointestinal side effects, 5% headache and other side effects were even rarer [7, 8, 31]. High rates among our participants may be explained by selection bias whereby people with stronger opinions, possibly attributable to having experienced side effects, were more likely to participate in our study. Another explanation could be that people are likely to develop side effects as a statistical probability when taking antivirals frequently or over a prolonged time, such as during the hyperepidemic context of the current AI outbreak.

We also observed a link between participants’ perceptions of their colleagues’ antiviral uptake and their views on antiviral use. Participants who reported that they did not take antivirals also reported that their colleagues had negative experiences taking antivirals, in line with a study finding that discussing antivirals with someone who had not experienced side effects was a facilitator for adherence, and vice versa [30]. Despite this, the same participants whose colleagues had had negative experiences reported that their decision to take antivirals was for personal reasons and not affected by their colleagues’ stories. The role of social influences in medication uptake is well-established, but considering the nuanced nature, future research should explore the role of social influence on antiviral uptake in occupational groups in an outbreak context.

Both exposed individuals and public health professionals raised concerns about the prolonged or frequent use of antivirals. Specifically, they questioned whether regular use leads to tolerance or contributes to antimicrobial resistance, reducing antivirals’ efficacy in the future. Previous clinical, in-vitro and modelling studies have found that the transmission of oseltamivir-resistant influenza strains is rare because neuraminidase mutations – the enzyme oseltamivir inhibits – reduce the fitness of the virus [32, 33]. Nevertheless, caution is warranted because influenza viruses, including AI, mutate frequently, and community transmission of oseltamivir-resistant influenza has been reported previously [34]. Therefore, limiting the provision of antivirals to those at the highest risk supports the prudent use of antivirals and addresses concerns about evolutionary pressures leading to oseltamivir-resistant strains.

We also found evidence of access-related barriers, highlighting the importance of making public health response more accessible and responsive to the needs of exposed individuals. As AI outbreak response was physically and mentally demanding, many preferred the provision of antivirals on or close to the exposure site. It is well-established that convenience is critical for behaviour change [35, 36], and convenient access has previously led to higher influenza vaccination uptake in occupational contexts, including among poultry workers [37, 38]. Therefore, convenient on-site provision of antivirals should be considered, especially in situations involving many exposed individuals.

Furthermore, HPPs often had challenges reaching exposed individuals on the phone, particularly as they were working long shifts on site wearing PPE which meant they were only contactable early morning or late evening. On the other hand, many frequently exposed individuals found repeated contact to be occasionally bothersome, particularly when it was attempted at unsuitable times, underscoring the need for communication that is timely yet sensitive to the realities of workers’ schedules. Similarly, as some professionals were said to have language barriers, it is essential that public health professionals provide materials in languages and formats suitable for the diverse workforce responding to AI to ensure they can make informed decisions about protecting themselves. Scalable, text-based illness monitoring systems can provide an efficient, accessible alternative that could complement more involved approaches for those who need or prefer it. These systems have demonstrated success in alleviating logistical burdens on public health teams and exposed individuals [39, 40], when tailored to preferred languages, can offer timely symptom monitoring that respects both the availability and accessibility needs of exposed individuals. Implementing such systems in future outbreaks could increase engagement with protective health measures, supporting a more effective and inclusive public health response.

Taken together, these findings have three main implications for practice. First, it is important for public health and healthcare professionals to advise how to minimise or manage side effects to support adherence and uptake when indicated in the future. Second, it is important to address the language barrier to enable people with non-English language preferences to make informed decisions. Third, public health guidance should carefully consider the risk‒benefit ratio of prophylactic antivirals when advised at scale. In other words, we suggest that public health response prioritises those at most risk of catching AI, such as those with significant exposure without sufficient protective measures or those vulnerable for other reasons. As noted above, since we conducted this study, guidance has been updated to recommending antivirals if complete PPE was not worn – although this continues to be interim guidance [23]. It is also possible this change may have reduced previously identified barriers to antiviral uptake, such as potentially reducing anticipated or experienced side effects among those frequently exposed and concerns about frequent and extended medication use.

Our study is the first to offer detailed insight into the factors affecting actual, not intended, uptake and adherence following exposure to AI. Whilst the focus groups with public health professionals enabled us to incorporate broader perspectives and triangulate self-reported factors affecting uptake, we could not recruit the professionals at most risk of AI infection to our study despite considerable efforts. This includes the catchers, cullers, and farm workers who often face additional challenges adhering to protective measures and accessing health services due to non-English language preferences and social and geographical isolation, limiting our understanding of the factors affecting uptake and the transferability of findings to these professional groups and people with non-English language preferences. Therefore, a subsequent project co-producing AI materials with people working on infected premises built on the connections and insights gained from this study and was able to involve catchers whose first languages were Polish and Bulgarian [41]. The contributions of participants who shared their views through an interpreter differed from those who spoke English, highlighting the importance of developing long-term mutually beneficial relationships between farming communities and public health professionals to be able to involve seldom-heard groups in work that affects them. Finally, due to practical constraints, the data analysis was solely conducted by the lead author. To mitigate this limitation, we have provided illustrative quotes and involved a team with diverse disciplinary backgrounds and job roles in interpreting the findings and ensuring that alternative viewpoints were considered. Furthermore, the triangulation of data from exposed individuals and public health professionals enabled the validation and cross-referencing of our findings.

A list of recommendations for practice is presented in Table 3. The recommendations are mapped to the findings in Supplementary material 3.

**Table 3:** Recommendations for practice

Recommendations
**Improving access to antivirals**:
• Make access to antivirals easy by providing them on-site or arranging for collection close to home for those at highest risk.
• Enable access to translated materials and interpreters for people with non-English language preferences.
• Provide materials in preferred formats (videos, easy-reads) and languages (e.g., Romanian, Bulgarian, Russian, Polish).
**Building relationships with exposed individuals**:
• Facilitate informed decision-making about the risks and benefits of taking antivirals.
• Build intermediaries’ skills (for example, culling team leads) to facilitate informed decision-making about antivirals.
• Develop alternative ways to contact exposed individuals, such as via their organisations and team leaders.
**Communication strategies**:
• Proactively provide information on what antivirals are, why they are prescribed, differences between prophylaxis and treatment, and how to minimise side effects. Check the individual’s understanding and correct identified misunderstandings and misperceptions (particularly regarding side effects).
• Communicate that antivirals are strongly recommended for those at highest risk.
• Inform exposed individuals about the pandemic potential of AI.
• Advise how to minimise or manage side effects to support uptake and adherence (e.g. taking the medication with food, contacting a pharmacist or a GP for advice on managing side effects).
• Raise awareness about AI symptoms in humans and who to contact when AI symptoms are suspected.
• Address concerns about long-term antiviral usage.
• Inform exposed individuals about the importance of finishing the prescribed course.
• Inform exposed individuals that PPE doesn’t offer 100% protection.
• Encourage exposed individuals to share positive or neutral experiences with antivirals to balance unfavourable experiences.
• Reinforce public health advice following repeated exposures.
**PPE usage**:
• Continue encouraging and enabling appropriate PPE usage.
• Encourage people to report PPE breaches.
**Risk-benefit considerations**:
• Public health guidance should carefully consider the risk-benefit ratio of prophylactic antivirals when advised at scale.
• Prioritise those at most risk of catching AI, considering exposure and vulnerability.
• Explore the availability of antivirals with fewer side effects.
**Sustained uptake and adherence**:
• Public health guidance should carefully consider the risk-benefit ratio of prophylactic antivirals to facilitate sustained uptake and adherence.
• Advise how to minimise or manage side effects to support uptake and adherence (e.g. taking the medication with food, contacting a pharmacist or a GP for advice on managing side effects).
• Make continued access to antivirals easy for those at highest risk (e.g. for extended exposures).

## Conclusion

We found factors that affect uptake among professionals exposed to AI to be multifaceted, including cognitive, motivational and practical factors. Public health interventions aiming to increase the uptake of antivirals should prioritise those at most risk and address multiple components of behaviour, such as advising how to manage side effects and addressing concerns about long-term usage and convenient access to antivirals for those at most risk.

## Electronic supplementary material

Below is the link to the electronic supplementary material.

**Supplementary Material 1:** Discussion guide for interviews with exposed individuals.

**Supplementary Material 2:** Discussion guide for focus groups with public health professionals.

**Supplementary Material 3:** Recommendations for practice mapped to findings.

## Data Availability

The data that support the findings of this study are available from UK Health Security Agency, but restrictions apply to the availability of these data, which were used under licence for the current study and so are not publicly available. The data are, however, available from the authors upon reasonable request and with the permission of UK Health Security Agency.

## Declarations

### Disclaimer

The views expressed are those of the author and not necessarily those of the UKHSA, NIHR, or the Department of Health and Social Care.

## Abbreviations

AI: Avian influenza
APHA: Animal and Plant Health Agency
HPP: Health Protection Practitioner
HPT: Health Protection Team
PPE: Personal protective equipment
REGG: Research Ethics Governance Group
UKHSA: United Kingdom Health Security Agency
