# Supplementary material for: “I definitely cannot afford to be feeling poorly if there’s no need to be”: a qualitative evaluation of antiviral uptake following suspected occupational exposure to avian influenza
Source: BMC Public Health. 2025 Feb 2;25:421. doi: 10.1186/s12889-025-21459-3 (PMC11789283; doi:10.1186/s12889-025-21459-3)
Supplement: Supplementary file 1 — Supplementary Material 1: Discussion guide for interviews with exposed individuals. [file 12889_2025_21459_MOESM1_ESM.docx]

**Supplementary material 1. Interview guide with exposed individuals.**

**General background**

1. **Thank you for taking part in this interview about your experiences with *bird / avian* flu and thoughts on antivirals. Just to start off, could you tell me briefly about your job role?**
   - If changed jobs, the role during which you were exposed to *bird / avian* flu
   - How long *have/did* you work there?
   - What do you do on daily basis?

**Knowledge about *bird / avian* flu and antivirals**

1. **Next, I’d like to ask a few questions about *bird / avian* flu and antivirals. Could you tell me briefly what do you know about *bird / avian* flu in humans?**
   - How ill do people get when they have *bird / avian* flu?
   - How does *bird / avian* flu spread?
   - How common is *bird / avian* flu in humans?
   - How vulnerable do you consider yourself to be to *bird / avian* flu?
   - What are the symptoms?
2. **Why do you think antivirals are being advised to people who are exposed to *bird / avian* flu?**
   - How effective are they in preventing people getting ill with *bird / avian* flu?
3. **What information has … given you about antivirals medication for *bird / avian* flu?**
   - Your employer (employer related to *bird / avian* flu exposure)
   - Your colleagues
   - Your friends and family
   - Have you received any training on *bird / avian flu* and antivirals?

**Exposure situation**

1. **I’d now like you to think back to the time when you were advised to take antiviral medication because of your exposure to *bird / avian* flu. You might have heard this medication being called Tamiflu or Osetamivir. First, could you tell me briefly about the activities you were doing at the time?**
   - Did you have direct contact with infected birds?
   - What PPE did you wear?
   - Were antivirals recommended for treatment, pre-exposure or post-exposure prophylaxis (prevention before exposure happened or after the exposure)?
2. **Did you feel you were at risk of catching *bird / avian* flu?**
   - If yes, why?
   - If no, why not?

**Experience of being advised to take antivirals**

1. **I’d now like you to think about the interactions you had with health professionals at the time you were exposed to *bird / avian* flu. Who contacted you to talk about your exposure?**
   - Were you contacted at a time that suited you?
   - If repeated contact, were you contacted by the same person or different people?
   - How long after your exposure were you contacted?
2. **Now, let’s think back at the conversation more specifically. How did they advise you to take antiviral medication?**
   - What information did they give you about *bird / avian* flu?
   - What information did they give you about antiviral medication?
   - Do you feel you had all the information you needed to make an informed decision about whether to take or not take antivirals?
   - Is there any other information that might have been useful?
   - Were you advised how to reduce or manage any side effects from the medication?
   - Did the way you were advised to take antivirals influence your decision to *take/not take antivirals*?
3. **If refused: What were your reasons for refusing antivirals?**
   - Was there anything you were particularly concerned about? (e.g., safety, side effects, ability to work)
   - How easy or difficult would it have been for you to get the medication?
   - Was there anything that could have been done that would have encouraged you to get the medication? (e.g., support, easier access to antivirals, better information)
   - Were there any reasons you thought you should get antivirals?
   - Are there any circumstances when you would take antivirals? (e.g., higher level exposure, symptomatic)

**If accepted: What were your reasons for taking antivirals?**

- - How easy or difficult was it for you to get the medication?
  - Was there anything you were particularly concerned about? (e.g., safety, side effects, ability to work)
  - Were the people around you supportive of your decision to take antivirals?

1. **If accepted: How was your experience taking antivirals?**
   - How long did you take antiviral medication? (e.g., did they finish the full course)
   - Was there anything that made it difficult to complete the course? (e.g., side effects, forgetting to take it, frequency, being busy/distracted)
   - How important was it to you to take antiviral medication as prescribed?

**Social context**

1. **We’ve already talked about quite a few things that influenced your decision to *(not)* take antivirals, so let’s think a bit more widely now about other people who are also exposed to *bird / avian flu*. Do you think people you work with have had antivirals when they were advised to take them?**
   - If not, why not?
   - If yes, does that affect your thoughts on taking antivirals?
   - It might be difficult to generalise but what’s the general sentiment on antivirals amongst the people you work with? (e.g., agreement, divisive issue, personal issue)
2. **In your opinion, is it important that people in your line of work take antivirals when advised to do so?**
   - If not, why not?
   - If yes, why?

**Improving uptake of antivirals**

1. **What do you think would increase the uptake of antivirals amongst people you work with?**
   - Are there any services, training, information, or anything else that you think could increase antiviral uptake?
   - Are there any organisations that could support this?
   - How can we support you better?
2. **We’ve come to the end of the interview now. Is there anything you want to add that we haven’t discussed?**
