# Supplementary material for: “I definitely cannot afford to be feeling poorly if there’s no need to be”: a qualitative evaluation of antiviral uptake following suspected occupational exposure to avian influenza
Source: BMC Public Health. 2025 Feb 2;25:421. doi: 10.1186/s12889-025-21459-3 (PMC11789283; doi:10.1186/s12889-025-21459-3)
Supplement: Supplementary file 2 — Supplementary Material 2: Discussion guide for focus groups with public health professionals. [file 12889_2025_21459_MOESM2_ESM.docx]

**Supplementary material 2. Topic guide for public health professional focus groups.**

**General background**

1. **I’d like to find out some more about you by going around the room one at a time. Could you tell me your first name and briefly how you’ve been involved in the avian flu response?**

**Knowledge about antivirals in avian flu**

1. **Could you tell me briefly why are antivirals being advised to people who have been exposed to avian flu under the Strict Approach?**
   - How effective are antivirals in preventing people getting ill with avian flu?
   - What are the risks of not taking antivirals when exposed to avian flu?
   - What are the benefits of taking antivirals because of exposure to avian flu for the 1) exposed individual 2) the general public?
   - In your opinion, how important is it that people exposed to avian flu take antivirals when advised to do so?
     - Does your opinion vary depending on the level of exposure?
2. **Where have you sought information about antivirals and avian flu?**
   - E.g., national guidance, local SOPs, research evidence
   - Do you feel like you know enough about why antivirals are advised?
   - What additional information would be helpful?

**Experiences of advising antivirals for asymptomatic individuals pre and post exposure**

1. **I’d now like to discuss the interactions you’ve had with exposed people. Could you describe a typical conversation with someone who has been exposed to avian flu?**
   - E.g., information given about avian flu, efficacy of antiviral medication, risk to the individual, risk to the public, making it easy for the individual
   - How do people usually respond to being advised to take antiviral medication?
   - Do you generally give advice on how to reduce or manage any side effects from the medication?
   - What approaches have been successful?
   - What have you found difficult in these conversations?
2. **In your experience, what are the main reasons people have accepted antivirals?**
   - E.g., feeling vulnerable, following a health recommendation, protecting others
   - How easy or difficult it is for people to get the medication?
3. **In your experience, what are the main reasons people have refused antivirals?**
   - E.g., not needed, social influences, fear of side effects, fear of not being able to work, cost
4. **How could we increase the uptake of antivirals amongst people exposed to avian flu at work?**
   - E.g., easier processes, different communications, tailored information

**Perceived barriers to arranging/prescribing antivirals**

1. **Is there anything that makes it difficult to arrange antivirals for exposed individuals?**
   - What are the steps you or your team need to take, so that the exposed person would receive antivirals once they have consented?
   - In your experience, has it been difficult arranging antivirals within the required timeframe?
   - Logistical barriers (e.g., clinical pathways, antiviral supplies/out of stock)
   - Practical (e.g., time available for actions, difficulties contacting exposed individuals/prescribers, language barrier)
   - Guidance
2. **What could UKHSA do to improve uptake of antivirals for people exposed to avian flu at work?**
   - E.g., improved processes, forms, reporting, more time available for actions, establishing better prescription pathways, changes to the guidance
   - Do you need additional training or support on avian flu?

**Closing questions**

1. **And just before we finish, and we will go round the table with this one, suppose that you were in charge and could make one change that would improve the avian flu response related to antivirals. What would you do?**
2. **We’ve talked about *(summarise the key themes).* Is there anything else you’d like to add?**
