# Supplementary material for: “I definitely cannot afford to be feeling poorly if there’s no need to be”: a qualitative evaluation of antiviral uptake following suspected occupational exposure to avian influenza
Source: BMC Public Health. 2025 Feb 2;25:421. doi: 10.1186/s12889-025-21459-3 (PMC11789283; doi:10.1186/s12889-025-21459-3)
Supplement: Supplementary file 3 — Supplementary Material 3: Recommendations for practice mapped to findings. [file 12889_2025_21459_MOESM3_ESM.docx]

**Supplementary material 3. Recommendations for practice mapped to findings.**

| **Research question** | **Results** | | | **Recommendations** |
| --- | --- | --- | --- | --- |
| **1. Interactions between public health professionals and exposed persons** | Not linked to COM-B | - HPT contact was reassuring for those with infrequent exposure - HPT contact was seen to be repetitive for those with frequent exposure - HPTs preferred on-site contact due to difficulties contacting exposed individuals - Public health professionals preferred contact through an intermediary over no contact - Need for accessible and engaging materials | | - Make access to antivirals easy by providing them on-site or arranging for collection close to home for those at highest risk. - Enable access to translated materials and interpreters for people with non-English language preferences. - Build intermediaries’ skills (for example, culling team leads) to facilitate informed decision-making about antivirals. - Develop alternative ways to contact exposed individuals, such as via their organisations and team leaders. - Provide materials in preferred languages (e.g., Romanian, Bulgarian, Russian, Polish) and formats (videos, easy-reads). |
| **2. Factors affecting uptake of antivirals** | **Capability**  The capacity to engage in the behaviour | **Psychological**  Capacity to engage in necessary thought processes | Exposed individuals had varied knowledge of avian influenza | - Inform exposed individuals about the pandemic potential of AI. - Raise awareness about AI symptoms in humans, and their consequences. |
|  |  |  | Exposed individuals had limited knowledge of antivirals | - Proactively provide information on what antivirals are, why they are prescribed, differences between prophylaxis and treatment, and how to minimise side effects to exposed individuals, including APHA staff and contractors, poultry industry leaders, and other people likely to be exposed to AI at work. Check the individual’s understanding and correct identified misunderstandings and misperceptions (particularly regarding side effects). |
|  |  |  | Exposed individuals had in-depth knowledge about risk mitigation | - Raise awareness about who to contact when AI symptoms are suspected. |
|  | **Motivation**  Brain processes that energise and direct behaviour | **Reflective**  Evaluation and plans | Exposed individuals reported following public health advice during their first exposures | - Reinforce public health advice following repeated exposures. |
|  |  |  | Exposed individuals conducted own risk assessments | - Facilitate informed decision-making about the risks and benefits of taking antivirals for the individual, their close contacts and the general population. |
|  |  |  | Feeling safe in PPE influenced risk perceptions | - Continue encouraging and enabling appropriate PPE usage. - Inform exposed individuals that PPE doesn’t offer 100% protection. - Encourage individuals to report PPE breaches. |
|  |  |  | Taking antivirals seen a personal choice | - Facilitate informed decision-making about the risks and benefits of taking antivirals for the individual, their close contacts and the general population. |
|  |  |  | Widespread concerns about long-term use among exposed individuals and public health professionals | - Address concerns about long-term use. - Public health guidance should carefully consider the risk-benefit ratio of prophylactic antivirals when advised at scale (e.g. those with significant exposure without sufficient protective measures or those vulnerable due to other reasons). |
|  |  |  | Exposed individuals were open to using antivirals in the future if they become more vulnerable, the virus changed or when strongly advised by a health professional | - For those at highest risk (e.g. due to significant unprotected exposure, existing health conditions), communicate that antivirals are strongly recommended. |
|  |  |  | Side effects were the biggest barrier to uptake | - Advise how to minimise or manage side effects to support uptake and adherence (e.g. taking the medication with food, contacting a pharmacist or a GP for advice on managing side effects). - Correct misperceptions about side effects when raised. - Explore the availability of other antivirals that have less side effects. - Public health guidance should carefully consider the risk-benefit ratio of prophylactic antivirals when advised at scale to facilitate sustained uptake and adherence. |
|  |  | **Automatic**  Emotions and impulses | Exhausting outbreak management necessitates easy access to antivirals | - Make access to antivirals easy by providing them on-site or arranging close to home for those at highest risk |
|  | **Opportunity**  Factors lying outside the individual that act as barriers or promoters of behaviour | **Social**  Cultural milieu that affects what we think about things | The role of social influences on antiviral uptake was not clear | - Address misperceptions by informing APHA staff, APHA contractors and leaders in the poultry industry about antivirals and how to minimise side effects. - Encourage exposed individuals to share positive or neutral experiences with antivirals to balance unfavourable experiences. |
|  |  | **Physical**  Physical opportunity in the environment | Easy access to antivirals was an important facilitator (e.g. close to infected premises, at a community pharmacy) | - Make access to antivirals easy by providing them on-site or arranging close to home for those at highest risk |
|  |  |  | Contraindications can be a barrier | - N/A |
| **Non-adherence with antivirals** | **Motivation** (brain processes that energise and direct behaviour) | **Automatic**  Emotions and impulses | Side effects were the biggest barrier to adherence | - Public health guidance should carefully consider the risk-benefit ratio of prophylactic antivirals when advised at scale to facilitate sustained uptake and adherence. |
|  | Not mapped to COM-B | | Evidence of frequent non-adherence with antivirals among exposed individuals | - Inform exposed individuals why finishing the prescribed course is important. - Encourage exposed individuals to return unused antivirals to the pharmacy. |
